# Erythema nodosum as a marker for objective disease activity in inflammatory bowel disease

**DOI:** 10.1093/crocol/otag013

**Published:** 2026-02-20

**Authors:** Solomon Sasson, Itay Kalisky, Gregory Rosenfeld, Jeremy Liu Chen Kiow, Brian Bressler

**Affiliations:** University of British Columbia, Vancouver, Canada; Division of Gastroenterology, The IBD Centre of British Columbia; Division of Gastroenterology, The IBD Centre of British Columbia; Division of Gastroenterology, Department of Medicine, Montreal University Hospital Centre (CHUM), Montreal, Quebec, Canada; Division of Gastroenterology, The IBD Centre of British Columbia

**Keywords:** erythema nodosum, extraintestinal manifestations, inflammatory bowel disease, endoscopic activity

## Abstract

**Background:**

Erythema nodosum (EN) is an inflammatory condition marked by tender, red nodules, typically on the extensor limbs. Though often linked to IBD activity, its association with objective markers like inflammatory labs or endoscopic findings remains unclear. This study examined the relationship between EN and objective inflammation in IBD patients.

**Methods:**

At a tertiary clinic with over 6500 patients, individuals with active EN were identified. Patients with active EN were identified, and symptomatic disease activity was assessed. Objective assessments included endoscopy, cross-sectional imaging (CT/MRI), fecal calprotectin, and CRP. Descriptive statistics compared objective and subjective disease activity in relation to EN.

**Results:**

Of 169 patients with documented EN, 95 had at least one objective assessment within our timeframe and were included. The mean age at EN presentation was 32 years; most had Crohn’s disease (80%) and were female (75%). Among CD patients, 93% had colonic involvement and 32% had penetrating disease. A total of 77 (74%) patients had gastrointestinal symptoms at the time of EN presentation, and 84 (81%) had objective evidence of active disease. Endoscopy was performed in 66% of cases, with 58 patients (84%) showing active inflammation. Among CD patients, 53% had mucosal ulcerations, while 67% of UC patients had severe disease (Mayo E3).

**Conclusions:**

In this cohort, most patients presenting with EN had both symptomatic and objective signs of intestinal inflammation. These findings support the use of objective testing in IBD patients with EN and suggest EN may indicate a more severe or complicated disease course.

## Graphical abstract

**Graphical Abstract:**
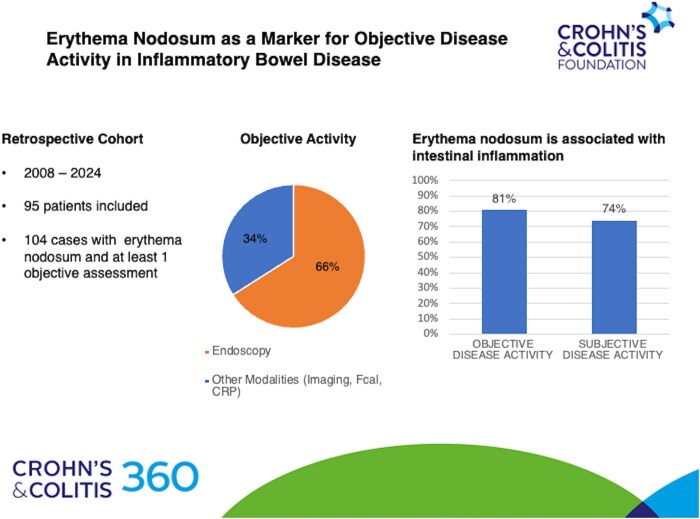

## Introduction

Extraintestinal manifestations (EIM) of inflammatory bowel disease (IBD) can significantly impact patients, contributing to morbidity and affecting quality of life beyond the primary gastrointestinal symptoms.^1^ EIMs occur in up to 40% of IBD patients and can involve the skin, joints, eyes, as well as other organs, underscoring the systemic nature of IBD.^2^

Erythema Nodosum (EN) is an inflammatory condition presenting as tender, erythematous nodules, primarily on the lower legs. These nodules typically range from 1 to 5 centimeters in diameter^3^ and are often accompanied by systemic symptoms such as fever, malaise, and joint pain, signaling an inflammatory response linked to both immune and non-immune conditions. Histologically, EN is classified as a type of panniculitis, marked by septal inflammation and edema without vasculitis, and is understood as a reactive cutaneous process driven by a type IV hypersensitivity response, often involving immune complex deposition.^4^ EN has a higher incidence in females than in males^5^ and is commonly associated with diseases including IBD, sarcoidosis, Behçet’s syndrome, systemic lupus erythematosus, infections, and certain medications (eg, oral contraceptives). Despite these associations, the underlying cause of EN remains undetermined in approximately 60% of cases.^6^

In the context of IBD, EN is recognized as the most common dermatologic manifestation, affecting 3%-10% of patients with ulcerative colitis (UC) and 4%-15% of those with Crohn’s disease (CD).^7^ Although EN is associated with IBD, the link between EN and intestinal disease activity is not fully understood.

The potential of EN as a noninvasive marker for IBD activity has attracted clinical interest, as it could provide a reliable indicator of underlying intestinal inflammation and guide therapeutic decisions. This relationship, however, has never actually been shown through objective measures of disease activity. Although recent ECCO guidelines state that EN is frequently associated with active IBD, this assertion is largely based on studies that assess symptomatic activity rather than objective inflammatory markers.^8^ A large Swiss cohort study^9^ involving 950 patients attempted to correlate EN with underlying IBD activity using validated scores based on patient-reported symptoms. Findings indicated that EN was reported in 6.8% of CD patients with an inactive disease and in 2.4% with an active disease, while in UC, EN was reported among 2% of patients with an inactive disease, and in 4.7% of those with an active disease. Nevertheless, these results were not statistically significant. Additionally, another study from the same cohort found that EN preceded intestinal disease in 14.3% of patients, a percentage comparable to that of pyoderma gangrenosum, an EIM which is not correlated with IBD activity.^10^ Dermatological EIMs may not always suggest active intestinal inflammation.

While gastrointestinal symptoms are typically used to assess disease activity in IBD, they are not always reliable indicators of mucosal inflammation.^11^ Subclinical inflammation can progress undetected without overt symptoms, leading to undertreatment and an increased risk of complications. The standard approach for assessing IBD activity combines clinical evaluation, laboratory markers like C-reactive protein (CRP) and fecal calprotectin (FCal), endoscopy and imaging assessment.

Given the limitations of symptom-based assessments, understanding whether EN reliably correlates with mucosal activity could provide a clinically valuable tool, by prompting objective evaluation and, if necessary, therapeutic intervention. This is particularly relevant for optimizing patient outcomes, as timely detection and treatment of flares can prevent complications and reduce healthcare costs associated with hospitalizations and surgeries.^12^

This study aims to investigate the association between EN and objective disease activity in IBD patients in a large Canadian IBD tertiary center.

## Methods

### Study design and setting

This retrospective study was conducted at the IBD Center of British Columbia—a tertiary, multi-disciplinary IBD clinic located in Vancouver, Canada. The center maintains a data registry containing over 6500 patient records extracted from the electronic medical records of the center. The clinical team includes four IBD specialists who routinely review patients from the center in case-based conferences to optimize care and minimize inter-observer variability. From January 1, 2008, to October 1, 2024, the data registry was searched to identify patients with EN. The study protocol followed predefined criteria to ensure comprehensive and standardized data collection, facilitating a thorough evaluation of the relationship between EN and IBD disease activity. The data was reviewed by two investigators to ensure accuracy and quality of data extracted.

### Patient selection

Eligible patients included were those with a documented history of CD, UC or IBD-unclassified, and a diagnosis of EN as recorded during clinical encounters. Identification of these patients was conducted in a two-step fashion:

First, we used the term “Erythema Nodosum” as the keyword search in Center’s IBD registry to screen for eligible patients. We then reviewed the patients’ electronic medical records to confirm accuracy of EN diagnosis and the documented date. Patients that had a missing date of their EN diagnosis were excluded. After screening, data from patients’ medical files at the time of EN presentation were retrieved, focusing on two primary domains: clinical symptoms and objective assessments of disease activity. Patients that were lacking data regarding their symptoms and/or objective assessments as per study protocol, were excluded.

### Clinical symptom assessment

To evaluate the presence of symptomatic disease activity, a binary approach was applied based on the physician’s assessment during each clinical encounter. This binary assessment served as a subjective measure of disease activity, capturing the clinician’s perspective at the time of presentation with EN on whether a patient was experiencing active IBD symptoms. A binary approach was chosen, as specific indices such as the Harvey Bradshaw Index or the Partial Mayo Score were not always recorded in patient’s files. Assessments were included if performed within 2 weeks before or after the EN presentation.

### Objective disease activity assessments

To evaluate the presence of objective disease activity, the following measures were assessed; endoscopy, imaging studies, and laboratory markers of inflammation. Endoscopy and imaging findings were included in the analysis if they had been performed within a 3-month window either before or after the presentation of EN. Simple

Endoscopic score for Crohn’s disease (SES CD) or the Mayo endoscopic score (MES) were used if available. The presence of ulcerations on endoscopy, defined as ulcers > 5 mm, were recorded as well. Imaging methods considered in the study included computed tomography (CT) and magnetic resonance imaging (MRI), all of which provided insights into mucosal and structural changes associated with disease activity. Laboratory values for CRP, erythrocyte sedimentation rate (ESR), and FCal, three markers commonly used to gauge inflammation in IBD, were also collected. These values were considered for analysis only if they were measured within a four-week period before or after EN presentation. Thresholds for active disease were defined as CRP > 5 mg/L, ESR > 30 mm/hr, and FCal levels > 250 µg/g. Objective assessments performed after EN presentation were included in the analysis only if no adjustments to medical management had been made in the intervening period. At our center, there is no standard protocol mandating routine investigations for all EN presentations. Evaluation of these patients follows usual IBD practice: the treating gastroenterologist performs a focused clinical assessment and orders testing when there is clinical suspicion of underlying intestinal inflammation. Endoscopy, defined as either colonoscopy or flexible sigmoidoscopy, was designated as the gold standard for assessing active disease in this study. Where endoscopy results were unavailable (whether unrecorded or not executed in the time frame outlined above), other modalities for objective assessment of disease activity as described above were used.

### Other parameters

Patients’ demographics, including sex, IBD subtype (CD/UC/IBD-U), IBD Montreal Classification, age at EN diagnosis, IBD-specific treatment at time of EN diagnosis, concomitant immune mediated disease, and presentation of other IBD-related EIM were collected. Laboratory parameters such as white blood cell count (WBC), hemoglobin, platelets, ferritin, and albumin were collected as adjuncts of inflammatory activity if taken in a 4-week window from EN presentation. In the case of EN recurrence, each event was presented as a separate case.

### Statistical analysis

Proportions, summarizing demographics and the prevalence of active versus inactive disease within the cohort were determined. The same calculations were applied to determine proportions of subjective disease activity based on clinical symptom assessment. Subsets were created for each diagnostic method—endoscopy, FCal, imaging studies, and CRP/ESR—to analyze the distribution of both subjective and objective disease activity according to the mode of investigation. Associations between categorical variables, including disease activity status and diagnostic modality, were analyzed using the one-sided Z test for a single proportion. For the test used, the null hypothesis assumed no correlation between disease activity (objective or subjective) to presentation of EN, while the alternative assumed a positive association between the two. A *P*< .05 was considered statistically significant. All statistical analyses were performed using standard statistical software to ensure computational accuracy.

### Ethical considerations

This retrospective study was approved by the University of British Columbia Research Ethics Board.

## Results

**Figure 1:**
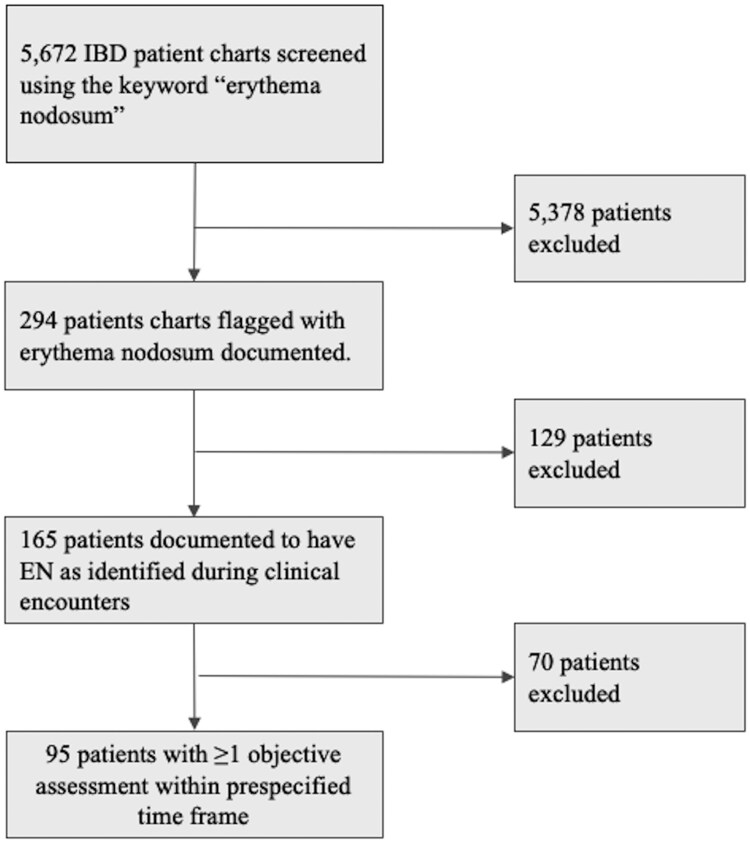
Inclusion and exclusion criteria flowchart.

Between January 1, 2008 and October 1, 2024, a total of 5672 patients were followed at the IBD Centre of BC. Among them, a total of 294 patients were identified in the IBD medical registry using the keyword “erythema nodosum” for initial screening. Of these, 165 patients were documented to have EN as identified during clinical encounters, representing roughly 2.9% of IBD patients. Finally, 95 patients had at least one objective assessment within the prespecified time frame and were included in the final analysis (Figure 1). Six patients experienced multiple episodes of EN during the study period, resulting in a total of 104 cases included in the final analysis. Amongst the patient population, 76 (80%) had CD, and 19 (20%) had UC. Baseline demographic and disease characteristics are summarized in Table 1. At presentation, 8 patients (8%) were receiving corticosteroids, 39 patients (38%) were on conventional therapy (5-ASA and/or Azathioprine), and 37 patients (35%) were being treated with biologic agents. Notably, 23 patients (22%) were not on active IBD therapy at the time of EN presentation, which primarily reflected cases where EN coincided with the initial diagnosis of IBD or instances where patients were between treatments at time of assessment. The cohort included both new-onset EN cases (70 cases, 67%) and recurrent EN cases (34 cases, 33%). Ten patients (10%) had EN coincide with their initial diagnosis of IBD. EN was confirmed on physical exam in 66 (63%) cases, whereas it was suspected in 38 (37%) cases. Suspected cases of EN were not confirmed on physical exam. A proportion of these diagnoses were made via telehealth based on the patient’s real-time description of the lesions on history-taking and, when available, review of patient-submitted photographs or video conference. Three patients were pregnant, and 10 (10%) patients were on combined oral contraception at presentation.

**Table 1:** Baseline characteristics of patients with IBD and EN.

Baseline characteristics	**Total (*n* = 104 cases** ^a^ **)**
**Age (years), median (IQR)**	32 (20-39)
**Sex, *n* (%)**	
** Male**	24 (25)
** Female**	71 (75)
**IBD subtype, *n* (%)**	95
** Crohn’s disease**	76 (80)
** UC**	19 (20)
**EN coinciding with initial diagnosis of IBD, *n* (%)**	10 (10)
**First diagnosis of EN, *n* (%)**	70 (67)
**Recurrence of EN, *n* (%)**	34 (33)
**Suspected EN, *n* (%)**	38 (37)
**Confirmed EN, *n* (%)**	66 (63)
**Immune-mediated comorbidities, *n* (%)**	
**Raynauds**	3 (3)
**Eczema**	6 (6)
**Pyoderma gangrenosum**	5 (5)
**Psoriasis**	5 (5)
**Hidradenitis suppurativa**	2 (2)
**Primary sclerosing cholangitis**	1 (1)
**IBD-associated arthropathy**	6 (6)
**Lichen-sclerosus**	1 (1)
**No medications, *n* (%)**	23 (22)
**Non-IBD medications, *n* (%)**	
** Oral contraception**	10 (10)
** Proton-pump inhibitors**	8 (8)
**IBD medications, *n* (%)**	
** Budesonide**	2 (2)
** Prednisone**	6 (6)
** 5-ASA**	29 (28)
** Azathioprine**	10 (10)
** Infliximab**	13 (12)
** Adalimumab**	12 (11)
** Ustekinumab**	7 (7)
** Vedolizumab**	5 (5)
**Crohn’s disease, *n* (%)**	
**Age at diagnosis**	
** A1**	33 (43)
** A2**	43 (57)
** A3**	5 (7)
**Disease location**	
** L1**	10 (13)
** L2**	32 (42)
** L3**	39 (51)
**Disease behavior^b^**	
** B1**	43 (57)
** B2**	18 (24)
** B3**	24 (32)
**Perianal involvement**	21 (28)
**Pouch**	2 (3)
**UC, *n* (%)**	
**UC disease location**	
** E1 (proctitis)**	2 (13)
** E2 (left-sided)**	3 (20)
** E3 (extensive)**	10 (67)

Abbreviations: EN, erythema nodosum; IBD, inflammatory bowel disease; IQR, interquartile range; UC, ulcerative colitis.

a Number of cases among a total of 95 patients.

b Overlap of disease behavior in the same patient is permissible.

Among the 104 cases who had at least 1 objective assessment within the specified timeframe, CRP/ESR results were available for 80 cases. Endoscopy was performed in 69 cases, imaging studies were conducted in 23 cases, and FCal measurements were obtained from 15 cases. The vast majority of CD patients had colonic involvement, with only 13% having isolated ileal disease. Thirty-two percent of CD patients had penetrating disease, and 24% had stricturing disease. Among UC patients, 53% of patients had extensive disease (E3), while only 2 patients had isolated proctitis.

Eighty-four cases (81%, CI 0.72-0.87, *P*< .0001) had at least one positive objective disease-activity assessment, while 77 (74%, CI 0.65-0.81, *P*< .0001) were deemed to have active disease based on their symptoms.

Using endoscopy as the gold standard, we performed a sub-analysis on the 69 cases who underwent endoscopic assessment for disease activity. Of these, 58 patients (84%, CI 0.73-0.91, *P*< 0.0001) demonstrated active disease, while 55 patients (80%, CI 0.69-0.87, *P*< 0.0001) reported subjective symptoms consistent with a flare. Among those with active disease on endoscopy, 31 out of 58 (53%) had mucosal ulcerations. The remaining 27 out of 58 (47%) had active disease without significant ulceration such as diffuse erythema, edema, friability, loss of vascular pattern, and/or erosions/aphthous ulcers. Additionally, 67% of patients with UC were classified as having severe endoscopic disease, defined by a MES of 3. Among 80 patients who had CRP levels taken, elevated CRP levels were found in 60 cases (75%, *P*< 0.0001), and they tended to be symptomatically active (74%, *P*< 0.0001). Further breakdowns of each diagnostic method are presented in Table 2. Notably, despite the smaller sample size, both imaging and FCal were also significantly abnormal among patients with EN.

**Table 2:** Subsets analysis of objective assessments.

	Total (*n*)	Objective	*P*-value
**Endoscopy subset**	69		
** Active**		58 (84%)	< .0001
** Inactive**		11 (16%)	
**Presence of ulcerations**	31(53%)		
**CRP (within endoscopy subset)**	46		
** Active (>5 mg/L)**		34 (74%)	
** Inactive (<5 mg/L)**		12 (26%)	
**Fecal calprotectin subset**	15		
** Active (FC >250 mcg/g)**		11 (73%)	< .05
** Inactive (FC < 250 mcg/g)**		4 (27%)	
**Imaging subset**	23		
** CT enterography**	18		
** MR enterography**	5		
** Active**		19 (79%)	< .05
** Inactive**		5 (21%)	
**CRP/ESR subset**	80		
** Active (CRP >5 mg/L)**		60 (75%)	< .0001
** Inactive (CRP < 5 mg/L)**		20 (25%)	

CRP, C-reactive protein; ESR, erythrocyte sedimentation rate.

Following presentation of EN, medication adjustment was conducted in the majority of cases (93, 89.5%) with the majority (44, 42%) commencing on a new corticosteroid course. 23% had their biologic regimen changed to a different drug or with dose escalation, and initiation of thiopurines for 17% of patients as shown in Table 3.

**Table 3:** Disease activity and treatment modifications at presentation.

Outcome	Total
**Subjective disease at presentation, *n* (%)**	77 (74)*
**Objective disease at presentation, *n* (%)**	84 (81)*
**Endoscopy**	69 (66)
**Fecal calprotectin**	15 (14)
**Imaging (CT, MRI)**	23 (22)
**CRP/ESR**	80 (77)
**Medication changes, *n* (%)**	
**New corticosteroid course**	44 (42)
**5-ASA initiation**	7 (7)
**Thiopurine initiation**	18 (17)
**Biologic treatment change**	21 (20)
**Biologic dose optimization**	3 (3)

Abbreviations: CRP, C-reactive protein; CT, computed tomography; ESR, erythrocyte sedimentation rate; MRI, magnetic resonance imaging.

* *P*<.0001.

## Discussion

Among 5672 patients with IBD followed at the IBD Centre of British Columbia, 165 patients were identified with a history of EN, representing 2.9% of the population. Of these, 110 (1.9%) had Crohn’s disease and 55 (1%) had UC. While the prevalence of EN in this cohort is lower than what has been mainly reported in the literature,^9^^,^^10^^,^^13^ other demographic distributions however are consistent, presenting significantly more common in women and in CD compared to UC.^9^^,^^10^^,^^13–15^ The decreased prevalence is probably due to less of a selection bias compared to other studies considering the way these patients were found using a database.

The IBD phenotype observed in this cohort differs from what is observed among the general IBD population.

Though the prevalence of those found with inflammatory and stricturing behavior was almost identical to a recently reported nationwide Canadian cohort,^16^ penetrating disease rate was much higher in our cohort. The vast majority of these patients exhibited colonic involvement, a pattern consistently reported in the literature in patients with EN.^13^ This pattern extends to patients with UC, with extensive pancolitis being the most common presentation, followed by left-sided colitis. This is consistent with previously reported studies.^13^^,^^14^

Among the 104 cases included in the analysis, 81% and 74% demonstrated evidence of active disease based on objective and subjective assessments, respectively. This observation was further reinforced when applying a subset analysis of patients who have solely undergone endoscopy to determine disease activity. Eighty-four percent of these patients had active disease using the gold standard for detection of inflammatory activity, with 80% of the patients in this subset analysis having gastrointestinal symptoms. All of these results were found to be statistically significant. While Vivraki et al. found no increase in the likelihood of a symptomatic IBD flare in the presence of active EN,^9^ our results are consistent with previously reported associations between EN presence and subjective activity. Moreover, our observation highlights the potential link between EN and mucosal inflammation. In fact, endoscopy revealed significant inflammation in both CD and UC as evident by the large proportions of patients having mucosal ulcerations.

To our knowledge, this is the first study specifically examining the association between IBD activity and EN using prespecified, validated objective assessments. While Veloso et al.^7^ found in a prospective study that EN was accompanied with active bowel disease in 88% of patients, it was not clear if that figure refers to objective activity, subjective activity, or both. Moreover, there was no time frame provided with regard to EN presence and objective assessment done. In our study we used not only readily available and validated tools to assess disease activity, but also limited their use for pre-specified timeframes to strengthen accuracy of our results.

This study has several limitations. As a retrospective cohort study, it is inherently limited in its ability to establish causal relationships. Additionally, there is potential for selection bias, as the inclusion criteria required patients to have at least one objective assessment. This may have inadvertently excluded individuals with milder disease or those managed without objective testing, thereby overrepresenting patients with more severe IBD and limiting the generalizability of our findings. Furthermore, the prespecified time window for objective and subjective assessments could have introduced additional bias. The broad definition of subjective disease activity is a limitation; only 6 and 16 cases had recorded Partial Mayo Scores and HBI scores, respectively, which may affect the interpretation and reliability of the subjective disease activity data. In addition, about one-third of the cohort were not confirmed to have EN on physical examination but rather had a suspicion of EN. Finally, information bias is another limitation to consider when dealing with retrospective studies, further limiting generalizability of results retrieved. Nevertheless, demographic characteristics such as female predominance, higher prevalence among CD patients and colonic pattern are aligned with what is reported in the literature, emphasizing credibility of our cohort.

In summary, our findings suggest that EN is an important marker of active inflammation regardless of the presence of gastrointestinal symptoms.

This study offers valuable insights into the association between EN and disease activity in IBD, underscoring the importance of incorporating objective assessments in clinical evaluation. The use of endoscopy as a gold standard strengthens the temporal relationship observed between EN and mucosal disease activity. Future studies evaluating the relationship of other known EIM of IBD and objective disease activity are needed.

## Data Availability

The data that support the findings of this study are available from IBD Centre of British Columbia, but restrictions apply to their availability, as they contain information from clinical patient records. Data are available from the authors upon reasonable request and with permission from the IBD Centre of British Columbia.
